# Supplementary material for: Resolving the full spectrum of human genome variation using Linked-Reads
Source: Genome Res. 2019 Apr;29(4):635–45. doi: 10.1101/gr.234443.118 (PMC6442396; doi:10.1101/gr.234443.118)
Supplement: Supplemental Material [file supp_gr.234443.118_Supplemental_longranger-2.2.zip › longranger-2.2.tar.bz2!/lr2.2-public/lib/stan/cmdstan/stan/doc/api/html/structstan_1_1gm_1_1matrix__var__decl-members.html]

Stan: Member List


|  |  |
| --- | --- |
|  | Stan  2.5.0  probability, sampling & optimization |


- Main Page
- Related Pages
- Namespaces
- Classes
- Files

- Class List
- Class Index
- Class Hierarchy
- Class Members

All Classes Namespaces Files Functions Variables Typedefs Enumerations Enumerator Friends Macros Pages

- stan
- gm
- matrix\_var\_decl

stan::gm::matrix\_var\_decl Member List

This is the complete list of members for stan::gm::matrix\_var\_decl, including all inherited members.

|  |  |  |
| --- | --- | --- |
| base\_type\_ | stan::gm::base\_var\_decl |  |
| base\_var\_decl() | stan::gm::base\_var\_decl |  |
| base\_var\_decl(const base\_expr\_type &base\_type) | stan::gm::base\_var\_decl |  |
| base\_var\_decl(const std::string &name, const std::vector< expression > &dims, const base\_expr\_type &base\_type) | stan::gm::base\_var\_decl |  |
| dims\_ | stan::gm::base\_var\_decl |  |
| M\_ | stan::gm::matrix\_var\_decl |  |
| matrix\_var\_decl() | stan::gm::matrix\_var\_decl |  |
| matrix\_var\_decl(range const &range, expression const &M, expression const &N, std::string const &name, std::vector< expression > const &dims) | stan::gm::matrix\_var\_decl |  |
| N\_ | stan::gm::matrix\_var\_decl |  |
| name\_ | stan::gm::base\_var\_decl |  |
| range\_ | stan::gm::matrix\_var\_decl |  |


---

[ Stan Home Page ]

*© 2011–2014,
Stan Development Team.*
